# No obesity paradox in patients with community-acquired pneumonia – secondary analysis of a randomized controlled trial

**DOI:** 10.1038/s41387-022-00190-7

**Published:** 2022-03-23

**Authors:** Angel N. Borisov, Claudine A. Blum, Mirjam Christ-Crain, Fahim Ebrahimi

**Affiliations:** 1 Department of Internal Medicine, Division of Endocrinology, Diabetes and Metabolism, University of Basel Hospital, Basel, Switzerland; 2 Department of Endocrinology and Medical Oncology, Fondazione Maugeri, Pavia, Italy; 3 University Department of Medicine, Kantonsspital Aarau, Aarau, Switzerland; 4 Clarunis, University Center for Gastrointestinal and Liver Diseases, Basel, Switzerland

**Keywords:** Fat metabolism, Obesity, Metabolic syndrome, Nutrition, Weight management

## Abstract

**Background:**

Obesity is associated with an increased risk for several chronic conditions and mortality. However, there are data in support of beneficial outcome in acute medical conditions such as community-acquired pneumonia (CAP), termed “obesity paradox”. The aim of this study was to test the association of BMI with clinical outcomes in a large randomized clinical trial of patients hospitalized with CAP.

**Design and Methods:**

In total, 773 patients hospitalized with CAP were included in this study. Patients were stratified into four groups according to their baseline BMI (underweight <18.5, normal weight 18.5–25, overweight 25–30, and obese >30 kg/m^2^). The primary endpoint was time to clinical stability (TTCS). Secondary endpoints included 30-day mortality, ICU admission rate, CAP complications, and duration of antibiotic treatment.

**Results:**

BMI and TTCS had a U-shaped association with shortest TTCS among patients at an overweight BMI of 28 kg/m^2^. In patients with obesity, there was a trend towards reduced hazards to reach clinical stability when compared to patients with normal weight (HR 0.82; 95%CI, 0.67–1.02; *p* = 0.07). In underweight BMI group TTCS was prolonged by 1 day (HR 0.63; 95%CI, 0.45–0.89; *p* = 0.008). There was no difference in mortality or ICU admission rates between BMI groups (*p* > *0.05*). While in the underweight BMI group the total duration of antibiotic treatment was prolonged by 2.5 days (95%CI, 0.88–4.20, *p* = 0.003), there was no difference in patients with obesity.

**Conclusions:**

The overweight BMI group had shortest time to clinical stability. While underweight patients face adverse clinical outcomes, there is neither beneficial, nor adverse outcome in patients with obesity hospitalized for CAP.

**ClinicalTrials.gov** (registration no. NCT00973154).

## Introduction

Obesity is associated with an increased risk for a wide range of chronic conditions such as cardiovascular disease, diabetes mellitus, and cancer [1]. However, a growing body of data suggests that despite these detrimental long-term effects, obesity might be associated with a beneficial outcome in the context of acute severe conditions—a phenomenon which has been coined *“obesity paradox”*. In fact, a recent meta-analysis summarizing more than 1.5 million outcomes of patients with community-acquired pneumonia (CAP) demonstrated that those who were overweight or with obesity were at a lower mortality risk compared to normal and underweight, respectively [2].

In fact, there are several pathophysiological aspects in support of an *obesity paradox*: (i) an increased metabolic reserve providing greater resistance to the catabolic state during acute infectious diseases; [2, 3] (ii) obesity as a state of chronic low-grade inflammation may enhance immune response toward a stronger T helper type 1 cell activation; [4] (iii) chest wall obesity ‘straps’ the thoracic cavity which might reduce transpulmonary pressure-mediated lung damage in the context of pneumonia and/or mechanical ventilation; [5] and (iv) elevated leptin has been shown to increase macrophage phagocytosis, neutrophil chemotaxis, natural killer cell cytotoxicity, as well as B and T cell function [6].

Nevertheless, thus far most data on clinical outcomes of patients with pneumonia are based on observational studies which did not thoroughly account for bias, such as a higher rate of active smokers among underweight patient [7–10**]**, as well as cancer-related cachexia, both contributing to poorer outcomes for patients at lower BMI [7–9,11–13].

Hence, in this secondary analysis of a large randomized, placebo-controlled, multicenter trial of hospitalized patients with CAP, our aim was to investigate the effects of BMI on relevant clinical outcomes, taking all relevant confounding factors into consideration.

## Subjects and methods

This is a secondary analysis of an investigator-initiated, multicenter, parallel-group, randomized, double-blind, placebo-controlled trial involving patients of all CAP severities admitted to the emergency department. Detailed description of the study design has been published elsewhere [14]. The main study demonstrated a significant clinical benefit of adjunct corticosteroids in hospitalized patients with CAP. In brief, consecutively admitted patients were randomized to receive either 50 mg of prednisone or placebo for 7 days on top of their standard treatment regimen. Inclusion criteria were age 18 years or older and hospital admission with diagnosis of CAP. Within 24 h of admission informed consent was collected. All patients were treated according to the ERS/ESCMID guidelines adapted for Switzerland [15]. Baseline data included patient history, comorbidities, clinical variables, and all parameters required for the calculation of the pneumonia severity index (PSI).

The conduct of the trial adhered to the Declaration of Helsinki and Good Clinical Practice Guidelines, and ethical committees of all participating hospitals approved the study before patient recruitment. The trial was registered with ClinicalTrials.gov (registration no. NCT00973154).

The primary objective of the current study was to investigate the association of BMI on the time to clinical stability (TTCS; defined as the time to stabilization of vital signs at two consecutive measurements ⩾12 h apart). Secondary objectives included analyses on the associations of BMI on 30-day all-cause mortality, intensive care unit (ICU) admission rate, CAP complications (including recurrence, acute respiratory distress syndrome, empyema, nosocomial infections, and severe adverse events possibly related to CAP), length of hospital stay (LOS), and length of total and intravenous antibiotic treatment.

### Statistical analysis

Unless stated otherwise, categorical variables are expressed as number (percentage) and continuous variables as median (interquartile range (IQR)). To analyze the association of BMI with clinical outcomes, the intention-to-treat population was subdivided in 4 subgroups: (i) underweight (<18.5 kg/m^2^), (ii) normal weight (18.5–5 kg/m^2^), (iii) overweight (25–30 kg/m^2^), and (iv) patients with obesity (>30 kg/m^2^). Multivariate regression models were used to analyze associations between BMI classes and outcomes of interest. Unadjusted and adjusted estimates of the effect sizes and corresponding 95% confidence intervals were determined using either linear, logistic or Cox proportional hazards regressions, as appropriate (normal weight subgroup served as base reference). All multivariate models were adjusted for the same variables: randomized treatment (prednisone), age, gender, diabetes mellitus, chronic obstructive pulmonary disease (COPD), asthma, heart failure, hypertension, peripheral artery disease, renal insufficiency, neoplastic disease, smoking status, and PSI. The multivariable fractional polynomials interaction (MFPI) approach was used to investigate the modifying effects of the continuous variable BMI on TTCS and LOS, respectively.

All statistical analyses were performed using Stata version 14.2 (StataCorp, College Station, TX, USA) and tests were done at a two-sided 5% significance level with two-sided 95% confidence intervals.

## Results

### Patient Characteristics

In total, 802 eligible patients were randomly assigned to receive either prednisone or placebo for 7 days. After blinded post-randomization exclusion of protocol violators and patients retrospectively not meeting eligibility criteria, a total of 773 patients of whom data on body weight and BMI were available were included in this study. Baseline characteristics, stratified according to BMI subgroups are depicted in (Table 1**)**. At baseline, median age was 73 years, and most patients with CAP (62%) were male. Most patients were either normal weight (36.7%) or overweight (35.4%). Patients with obesity more often suffered from hypertension (69.2%) and had higher rates of diabetes mellitus (35.5%), compared to those of normal- or underweight. Among patients in the underweight group, highest proportions of smokers (32.6%) and cancer-affected individuals (13.0%) were observed. Patients from all four BMI groups had comparable severities of pneumonia according to the PSI score, with around half of the patients in the high-risk PSI classes IV and V.

**Table 1:** Baseline characteristics and clinical variables of enrolled patients.

Characteristic/variable	Total (*n* = 773)	Normal weight (*n* = 284)	Underweight (*n* = 46)	Overweight (*n* = 274)	Obese (*n* = 169)	*p* value
**General characteristics**						
Age, years	73 (61, 83)	73 (60, 83)	72 (54, 89)	75 (61, 83)	72.0 (61, 81)	0.72
Male sex	481 (62.2%)	160 (56.3%)	24 (52.2%)	198 (72.3%)	99 (58.6%)	<0.001
BMI, kg/m^2^	25.9 (23.0, 29.4)	22.8 (21.2, 23.9)	17.5 (16.7, 18.0)	27.4 (26.0, 28.7)	33.3 (31.2, 35.9)	<0.001
Body weight, kg	75 (65, 88)	65 (59, 72)	50 (45, 54)	80 (74, 87)	95 (89, 106)	<0.001
Smoking status	199 (25.7%)	84 (29.6%)	15 (32.6%)	61 (22.3%)	39 (23.1%)	0.13
Packyears, years	5 (0, 37)	5 (0, 37)	7 (0, 30)	7 (0, 40)	5 (0, 40)	0.97
**Comorbidities**						
Diabetes mellitus	152 (19.7%)	39 (13.7%)	1 (2.2%)	52 (19.0%)	60 (35.5%)	<0.001
Insulin treatment	51 (6.6%)	13 (4.6%)	0 (0.0%)	11 (4.0%)	27 (16.0%)	<0.001
COPD	133 (17.2%)	52 (18.3%)	15 (32.6%)	43 (15.7%)	23 (13.6%)	0.020
Asthma	46 (6.0%)	13 (4.6%)	1 (2.2%)	24 (8.8%)	8 (4.7%)	0.092
Heart failure	140 (18.1%)	42 (14.8%)	7 (15.2%)	53 (19.3%)	38 (22.5%)	0.18
Hypertension	412 (53.3%)	128 (45.1%)	22 (47.8%)	145 (52.9%)	117 (69.2%)	<0.001
Cerebrovascular disease	66 (8.5%)	24 (8.5%)	2 (4.3%)	23 (8.4%)	17 (10.1%)	0.67
Peripheral artery occlusive disease	47 (6.1%)	13 (4.6%)	6 (13.0%)	18 (6.6%)	10 (5.9%)	0.16
Renal insufficiency	248 (32.1%)	69 (24.3%)	15 (32.6%)	100 (36.5%)	64 (37.9%)	0.005
Neoplastic disease	53 (6.9%)	19 (6.7%)	6 (13.0%)	19 (6.9%)	9 (5.3%)	0.33
Steroid pretreatment	28 (3.6%)	14 (4.9%)	0 (0.0%)	9 (3.3%)	5 (3%)	0.70
Antibiotic pretreatment	176 (22.8%)	64 (22.5%)	8 (17.4%)	70 (25.5%)	34 (20.1%)	0.45
**Clinical variables**						
Days with symptoms, days	4 (2, 7)	4 (2, 7)	7 (3, 14)	4 (2, 7)	4 (2, 6)	0.007
Systolic blood pressure, mmHg	124 (110, 140)	120 (108, 136)	119 (103, 139)	126 (114, 140)	128 (114, 143)	0.001
Diastolic blood pressure, mmHg	69 (60, 78)	66 (58, 76)	65.5 (58, 73)	70 (60, 78)	70 (61, 80)	0.001
Pulse, bpm	84 (72, 95.5)	84 (76, 96)	83 (71, 100)	80 (70, 94)	84 (76, 95)	0.047
Respiratory frequency, breaths/min	20 (18, 24)	20 (17, 24)	22 (16, 24)	20 (18, 24)	20 (18, 25.5)	0.15
Temperature, °C [in-ear]	37.6 (37.0, 38.2)	37.5 (37.0, 38.1)	37.8 (37.0, 38.3)	37.6 (36.9, 38.3)	37.5 (37.0, 38.2)	0.79
SIRS, points.	2 (1, 3)	2 (1, 3)	2 (1, 3)	2 (1, 3)	2 (2, 3)	0.33
PSI classes^†^						
I, II and III	395 (51.1%)	150 (52.8%)	24 (52.2%)	136 (49.6%)	85 (50.3%)	0.89
IV and V	378 (48.9%)	134 (47.2%)	22 (47.8%)	138 (50.4%)	84 (49.7%)	0.89
PSI, points	89 (64, 113)	87 (63, 112)	89 (63, 119)	90.5 (68, 114)	90 (66, 112)	0.75
**Laboratory values**						
C-reactive protein, mg/L	161.4 (80.2, 249.0)	165.0 (93.8, 251.5)	177.5 (88.6, 259.7)	153.0 (79.1, 243.0)	170.0 (62.7, 249.0)	0.59
White blood cell count, G/L	12.1 (8.8, 15.6)	11.9 (8.5, 15.6)	12.1 (9.6, 15.3)	12.1 (8.4, 15.8)	12.1 (9.4, 15.8)	0.74
Fasting glucose, mmol/L	6.3 (5.5, 7.7)	5.9 (5.3, 7.3)	5.9 (5.6, 6.5)	6.5 (5.5, 8.0)	6.8 (5.8, 9.0)	<0.001

Data are presented as n, median (IQR) or n (%), unless otherwise stated. *BMI* body mass index, *COPD* chronic obstructive pulmonary disease, *SIRS* systemic inflammatory response syndrome, *PSI* Pneumonia Severity Index.; the PSI is a clinical prediction rule to calculate the probability of morbidity and mortality in patients with community-acquired pneumonia; PSI risk class I corresponds to age ⩽50 years and no risk factors (⩽50 points), risk class II to <70 points, risk class III to 71–90 points, risk class IV to 91–130 points and risk class V to >130 point.

### BMI and time to clinical stability

**Fig. 1:**
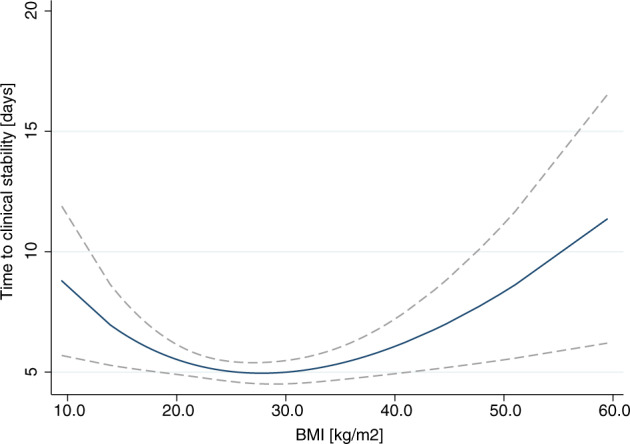
BMI and time to clinical stability. Fractional polynomial estimation of the association of BMI with the time to clinical stability (TTCS) (blue curve) along with the confidence interval of the mean (gray area). There is a U-shaped association between TTCS and BMI.

Shortest time to clinical stability was observed among patients with overweight [3.0 days (IQR 2.6–4.0)] and patients of normal body weight [3.4 days (IQR 3.0–4.0); *p* > 0.05]. TTCS was significantly prolonged in underweight [4.4 days (IQR 3.0–6.8)] and there was a trend towards prolonged TTCS in patients with obesity [4.4 days (IQR 3.5–5.0)] when compared to patients of normal body weight (Table 2). Multivariate Cox regression analysis revealed that hazards to reach clinical stability were significantly reduced in the underweight BMI group [HR 0.63, (95%CI, 0.45–0.89; *p* = 0.008)] and in patients with obesity [HR 0.82, (95%CI, 0.67–1.02; *p* = 0.07)], however in patients with obesity it did not reach statistical significance. Analyses of multivariable fractional polynomials interaction confirmed a U-shaped association between BMI and TTCS with shortest time to clinical stability at an overweight BMI between 27 and 29 kg/m^2^ (Fig. 1).

**Table 2:** Outcomes Absolute Values and Multivariate Cox regression analysis.

	Underweight (BMI < 18.5)	Normal weight (BMI 18.5–25)	Overweight (BMI 25–30)	Obese (BMI > 30)	Multivariable adjusted regression analysis OR, HR or regression coefficient (95% CI)					
					Underweight vs. Normal weight		Overweight vs. Normal weight		Obese vs. Normal weight	
**Primary outcome**					OR/HR	*P* value	OR/HR	*P* value	OR/HR	*P* value
^§^TTCS (days)	4.4 (3.0–6.8)	3.4 (3.0–4.0)	3.0 (2.6–4.0)	4.4 (3.5–5.0)	^#^0.63	0.008	^#^1.01	0.88	^#^0.82	0.07
**Secondary outcomes**										
LOS (days)	7.0 (7.0–11.0)	7.0 (6.0–7.0)	7.0 (6.0–7.0)	7.0 (6.0–8.0)	^#^0.55	<0.001	^#^1.00	0.96	^#^0.97	0.79
30-day Mortality	4 (8.7)	10 (3.5)	6 (2.2)	7 (4.1)	^¶^2.19	0.28	^¶^0.58	0.35	^¶^1.41	0.58
ICU admission	0	15 (5.3)	10 (3.7)	11 (6.5)	–	–	^¶^0.72	0.47	^¶^1.04	0.93
^ƒ^CAP complications	18 (39.1)	71 (27.3)	80 (31.5)	50 (31.9)	^¶^1.71	0.13	^¶^1.19	0.40	^¶^1.19	0.46
Intravenous antibiotics (days)	5.0 (3.0–8.0)	4.0 (3.0–6.0)	5.0 (3.0–6.5)	4.0 (3.0–6.0)	^+^1.47	0.049	^+^0.19	0.63	^+^−0.05	0.91
Total antibiotics (days)	10.0 (7.5–14.0)	8.0 (7.0–11.0)	9.0 (7.0-11.0)	9.0 (7.0–11.0)	^+^2.54	0.003	^+^−0.05	0.91	^+^0.05	0.93

Data are presented as *n*, *n* (%) or median (interquartile range), *ICU* intensive care unit, *TTCS* time to clinical stability, *LOS* length of hospital stay, *CAP* community-acquired pneumonia. #hazard ratio; ¶odds ratio; +regression coefficient; §TTCS defined as time to clinical stabilization of vital signs at two consecutive measurements ⩾12 h apart ƒ: CAP complications defined as recurrence, acute respiratory distress syndrome, empyema, nosocomial infections until day 30, or serious adverse events possibly related to CAP, ICU stay or readmission to hospital.

### BMI and length of hospital stay

**Fig. 2:**
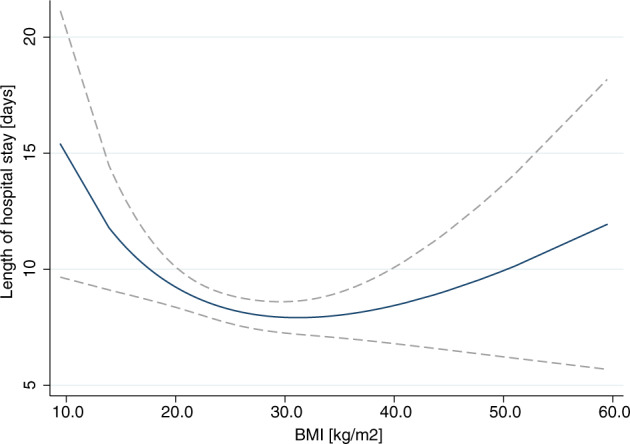
BMI and length of hospital stay. Fractional polynomial estimation of the association of BMI with the length of hospital stay (LOS) (blue curve) along with the confidence interval of the mean (gray area). There is a U-shaped association between LOS and BMI.

The median length of hospital stay for patients of all BMI subgroups was 7 days, (IQR for underweight 7–11 days, normal weight 6–7 days, overweight 6–7 days, and patients with obesity 6–8 days) (Table 2**)** with comparatively longest hospital stays in the underweight group. In multivariate cox regression analysis, the underweight BMI group had significantly reduced hazards for hospital discharge [HR 0.55 (95%CI, 0.40-0.76; *p* < 0.001] when compared to patients of normal weight (Table 2). Length of hospital stay, and BMI shared a U-shaped association with the shortest duration of hospitalization falling within a BMI range of 29 to 32 kg/m^2^ (Fig. 2).

### BMI and 30-day mortality & ICU admission rates

The greatest proportion of deaths within 30 days of admission was observed among the underweight BMI group (8.7%), which was more than twice as high compared to patients of normal body weight (3.5%).

There was a trend towards higher 30-day mortality rates in the underweight BMI group [OR 2.19 (95%CI, 0.53–9.00)] and lower mortality rates among overweight BMI [OR 0.58 (95% CI, 0.19–1.82)] (Table 2). However, multivariate cox regression analysis could not reveal significant differences between the BMI subgroups. Likewise, there was no difference in ICU admission rates between BMI classes (*p* > 0.05) (Table 2).

### BMI and duration of antibiotic treatment

Duration of IV antibiotic treatment was prolonged by around one day in patients falling in the underweight [5 days (IQR 3.0–8.0)] and overweight groups [5 days (IQR 3.0–6.5)] respectively, compared to patients of normal body weight [4 days (IQR 3.0–5.0)]. Multivariable adjusted regression analysis revealed an average prolongation of treatment by 1.47 days in the underweight BMI group (95%CI, 0.01–2.94; *p* = 0.049) (Table 2).

A longer total duration of antibiotic treatment was also observed among underweight BMI group with a median duration of 10 days (IQR 7.5–14), one day more than overweight and patients with obesity, that required 9 days (IQR 7–11), and patients with normal weight requiring only 8 days (IQR 7–11). Multivariate cox regression analysis revealed a between-group difference prolonged by 2.5 days for underweight BMI compared to patients of normal body weight (95%CI, 0.88–4.20, *p* = 0.003).

## Discussion

In this large randomized-controlled trial of patients hospitalized with CAP, a *U*-shaped relationship of BMI with clinical outcomes was observed—slight overweight was associated with shortest time to clinical stability. Our findings show that patients with obesity did not fare better than normal weight in community-acquired pneumonia, contrary to the hypothesis of an *obesity paradox*. While obesity per se was neither beneficial, nor associated with deleterious outcome, underweight was the strongest predictor for prolonged clinical instability, length of hospitalization, and duration of antibiotic treatment.

There are numerous studies of different patient populations suggesting a beneficial outcome among patients with obesity, termed *obesity paradox*: patients undergoing surgical procedures [16], patients on chronic hemodialysis [9, 17**]**, as well as patients hospitalized due to heart failure or myocardial infarction, respectively [11]. Comparable findings were reported for patients hospitalized with community-acquired pneumonia. A prospective observational study from the UK including more than thousand patients with CAP reported a reduced 30-day mortality rate among patients with obesity [12]. A similar study investigating a patient cohort in China, found a lower mortality rate among patients with obesity one year after hospitalization for CAP [18]. In line with that, a large Swiss cohort study observed improved survival rates in patients with obesity even 6 years after index hospitalization for CAP [19]. According to some studies mortality is lower in patients with obesity who develop acute respiratory distress syndrome (ARDS). Several pathophysiological aspects of obesity have been discussed as causative for this observation, such as (i) lower levels of cytokines (i.e., IL-6 & IL-8), (ii) larger number of phenotype M2 macrophages, which work antiinflammatory, (iii) as well as greater quantities of lipids/ lipoproteins that bind and inactivate endotoxins [20, 21].

In contrast to these results, we did not observe any differences in mortality between the pre-defined BMI subgroups. In our graphs (Figs. 1 and 2) overweight appears to have an advantage for time to clinical stability (TTCS) and length of hospital stay (LOS), both underweight as well as obesity were associated with an increased risk of adverse outcome, as mirrored by a U-shaped association of BMI and clinical outcomes. Interestingly, within this association the slopes towards lower BMI were steeper. This might be the result of stringent adjustment of analyses for all known confounding factors. In fact, previous results in support of an advantageous clinical outcome in patients with obesity were prone to have a high risk of confounding and reverse causation [8]. As clearly demonstrated by Stokes et al., results in support of an apparent “*obesity paradox*” were consistently confounded by smoking and inclusion of high-risk patients whose weight loss from chronic disease has put them within a normal or low BMI range. Most strikingly, as their results demonstrated, once the reference category was limited to patients with a stable normal weight and once the confounding factor of smoking was controlled for, obesity was associated with a significantly higher mortality risk [13]. Additionally, collider stratification bias (a greater risk of infection in patients with obesity increases the chance that more of their cases are mild [22]) and performance bias (higher rates of co-morbidities make it more likely for patients with obesity to be monitored by a health-practitioner and hence are treated earlier [7, 19**]**) may have produced favorable outcomes for patients with obesity in previous observational studies. Against this background, we designed our multivariate analysis, prioritizing the control for confounding factors.

Previous data showed that both extremes of the BMI spectrum increase the general susceptibility to infections [8, 23**]**. Indeed, immune function has been shown to be altered both in patients with obesity [24] and among patients with underweight and malnutrition [25, 26]. In obesity, adipokines secreted from adipose tissue have been shown to induce both pro- and anti-inflammatory effects on the immune system [27, 28].

While the effect of obesity on immune function remains under debate, the evidence of immune dysfunction in states of malnutrition and underweight is rather established [29–32]. In a review chronic caloric restriction significantly increased the susceptibility to infectious diseases due to a decrease in immune cell function and cellularity [27, 33]. This may indeed explain our findings of adverse outcome among underweight BMI. In line with our data, a recent systematic review assessing all possible risk factors for CAP in adult patients revealed that there was no effect of overweight, while in contrast a poor nutritional status was a strong predictor of CAP [34].

There are some potential limitations that need to be considered when evaluating the results of this study: First, we lack data on the temporal dynamics of body weight and composition in our cohort, therefore changes of body weight over time cannot be excluded. Second, our study predominantly represented the elderly, hence our results cannot be extrapolated to younger populations. Likewise, the study included only patients with CAP who required admission to hospital, therefore results do not apply to patients in ambulatory care. Finally, the study was not powered to investigate effects on mortality or outcomes of patients with ARDS. Nevertheless, this study has several strengths: a large and well characterized cohort with different severities of CAP representative for patients usually treated in emergency departments and hospitals. Assessment of clinically relevant outcomes including clinical stability which is a good marker of short-term outcome. Importantly, all results were meticulously controlled for the major confounders: smoking status, cancer and comorbidities associated with malnutrition.

In conclusion, our study cannot confirm an “*obesity paradox*”, while it underlines that underweight may aggravate the course of disease in patients hospitalized with CAP. Despite a nadir in TTCS and LOS for overweight within our MFPI graphs our multivariate regression analysis did not reveal an advantage in clinical outcome. To unveil whether patients with overweight enjoy any benefit, future studies should include more clinical aspects such as waist circumference, hip to waist ratio or fat mass in order to better reflect the metabolic status than BMI alone. Furthermore, mechanistic studies in this field are warranted investigating underlying mechanisms of obesity, immunometabolism and acute systemic infections.

## References

[1] MacMahon S, Baigent C, Duffy S, Rodgers A, Tominaga S, Chambless L (2009). Body-mass index and cause-specific mortality in 900 000 adults: Collaborative analyses of 57 prospective studies. Lancet.

[2] Nie W, Zhang Y, Jee SH, Jung KJ, Li B, Xiu Q (2014). Obesity survival paradox in pneumonia: A meta-analysis. BMC Med.

[3] King P, Mortensen EM, Bollinger M, Restrepo MI, Copeland LA, Pugh MJV (2013). Impact of obesity on outcomes for patients hospitalised with pneumonia. Eur Respir J.

[4] Falagas ME, Athanasoulia AP, Peppas G, Karageorgopoulos DE (2009). Effect of body mass index on the outcome of infections: A systematic review: Obesity Comorbidities. Obes Rev.

[5] Kahlon S, Eurich DT, Padwal RS, Malhotra A, Minhas-Sandhu JK, Marrie TJ (2013). Obesity and outcomes in patients hospitalized with pneumonia. Clin Microbiol Infect.

[6] La Cava A, Matarese G (2004). The weight of leptin in immunity. Nat Rev Immunol.

[7] Atamna A, Elis A, Gilady E, Gitter-Azulay L, Bishara J (2017). How obesity impacts outcomes of infectious diseases. Eur J Clin Microbiol Infect Dis.

[8] Roth J, Sahota N, Patel P, Mehdi SF, Wiese MM, Mahboob HB (2016). Obesity paradox, obesity orthodox, and the metabolic syndrome: An approach to unity. Mol Med..

[9] Weir MR (2011). Body mass index-mortality paradox in hemodialysis patients: Blood pressure, blood volume, and nutritional status. Hypertension.

[10] Albanes D, Jones Y, Micozzi MS, Mattson ME (1987). Associations between smoking and body weight in the US population: Analysis of NHANES II. Am J Public Health.

[11] Prescott HC, Chang VW (2018). Overweight or obese BMI is associated with earlier, but not later survival after common acute illnesses. BMC Geriatr..

[12] Singanayagam A, Singanayagam A, Chalmers JD (2013). Obesity is associated with improved survival in community-acquired pneumonia. Eur Respir J.

[13] Stokes A, Preston SH (2015). Smoking and reverse causation create an obesity paradox in cardiovascular disease. Obes.

[14] Blum CA, Nigro N, Winzeler B, Suter-Widmer I, Schuetz P, Briel M (2014). Corticosteroid treatment for community-acquired pneumonia - The STEP trial: Study protocol for a randomized controlled trial. Trials..

[15] Laifer G, Flückiger U, Scheidegger C, Boggian K, Mühlemann K, Weber R, et al. Management of CAP (ERS/ESCMID Guidelines adapted for Switzerland) Management of Community Acquired Pneumonia (CAP) in Adults (ERS/ESCMID guidelines 1 adapted for Switzerland). 2003:1–12.

[16] Utzolino S, Ditzel CM, Baier PK, Hopt UT, Kaffarnik MF (2014). The obesity paradox in surgical intensive care patients with peritonitis. J Crit Care.

[17] Calabia J, Arcos E, Carrero JJ, Comas J, Vallés M (2015). Does the obesity survival paradox of dialysis patients differ with age?. Blood Purif..

[18] Chen J, Wang J, Jiang H, Li MC, He SY, Li XP (2019). Lower long-term mortality in obese patients with community-acquired pneumonia: Possible role of CRP. Clinics.

[19] Braun N, Hoess C, Kutz A, Christ-Crain M, Thomann R, Henzen C (2017). Obesity paradox in patients with community-acquired pneumonia: Is inflammation the missing link?. Nutr.

[20] Umbrello M, Fumagalli J, Pesenti A, Chiumello D (2019). Pathophysiology and Management of Acute Respiratory Distress Syndrome in Obese Patients. Semin Respir Crit Care Med.

[21] Ni YN, Luo J, Yu H, Wang YW, Hu YH, Liu D (2017). Can body mass index predict clinical outcomes for patients with acute lung injury/acute respiratory distress syndrome? A meta-analysis. Crit Care.

[22] Sperrin M, Candlish J, Badrick E, Renehan A, Buchan I (2016). Collider bias is only a partial explanation for the obesity paradox. Epidemiology.

[23] Dobner J, Kaser S (2018). Body mass index and the risk of infection - from underweight to obesity. Clin Microbiol Infect..

[24] Milner JJ, Beck MA (2012). The impact of obesity on the immune response to infection. Proc Nutr Soc.

[25] Ritz BW, Gardner EM (2006). Malnutrition and energy restriction differentially affect viral immunity. J Nutr.

[26] Rytter MJH, Kolte L, Briend A, Friis H, Christensen VB (2014). The immune system in children with malnutrition - A systematic review. PLoS One.

[27] Carbone F, La Rocca C, De Candia P, Procaccini C, Colamatteo A, Micillo T (2016). Metabolic control of immune tolerance in health and autoimmunity. Semin Immunol.

[28] Ouchi N, Parker JL, Lugus JJ, Walsh K (2011). Adipokines in inflammation and metabolic disease. Nat Rev Immunol.

[29] Hotamisligil GS (2017). Inflammation, metaflammation and immunometabolic disorders. Nature.

[30] Veronese N, Cereda E, Solmi M, Fowler SA, Manzato E, Maggi S (2015). Inverse relationship between body mass index and mortality in older nursing home residents: A meta-analysis of 19,538 elderly subjects. Obes Rev.

[31] Christadoss P, Talal N, Lindstrom J, Fernandes G (1984). Suppression of cellular and humoral immunity to T-dependent antigens by calorie restriction. Cell Immunol.

[32] Yang L, Chan KP, Lee RSYIN, Chan WM, Lai HK, Thach TQ (2013). Obesity and influenza associated mortality: Evidence from an elderly cohort in Hong Kong. Prev Med (Balt).

[33] Wing EJ, Magee DM, Barczynski LK (1988). Acute starvation in mice reduces the number of T cells and suppresses the development of T-cell-mediated immunity. Immunology.

[34] Almirall J, Serra-Prat M, Bolíbar I, Balasso V (2017). Risk Factors for Community-Acquired Pneumonia in Adults: A Systematic Review of Observational Studies. Respiration..

